# Accuracy of artificial intelligence applications in periodontics: a thematic narrative review

**DOI:** 10.3389/fdmed.2026.1729825

**Published:** 2026-01-22

**Authors:** Ady Azhari

**Affiliations:** Department of Periodontology, Faculty of Dentistry, King Abdulaziz University, Jeddah, Saudi Arabia

**Keywords:** artificial intelligence, cone-beam computed tomography, diagnostic accuracy, intraoral imaging, machine learning, panoramic radiography, periodontal bone loss, periodontics

## Abstract

**Background:**

Artificial intelligence (AI) has been increasingly applied to periodontal diagnostics across periapical, bitewing, panoramic radiographs, cone-beam computed tomography (CBCT), and intraoral photographs. Recent multicenter, external validation, and explainability-focused studies have advanced the field, yet variability in datasets, anatomical sites, reference standards, model architectures, and reporting practices introduces significant heterogeneity. A structured synthesis of current evidence is therefore warranted.

**Main text:**

This review synthesizes 35 studies published between 2019 and 2025, evaluating AI applications in four diagnostic domains: detection of periodontal bone loss, measurement of alveolar bone levels, identification of furcation involvement, and detection of periapical lesions. Convolutional neural network (CNN)-based models using periapical radiographs achieved moderate-to-high diagnostic accuracy (0.82–0.85) and AUCs above 0.88, comparable to clinician performance. Panoramic radiographs yielded lower sensitivity and specificity than CBCT, where deep learning systems reached higher accuracy (up to 0.91) and superior volumetric assessment. Intraoral photographic analyses showed variable performance (0.46–1.00), largely due to inconsistent imaging and reference standards. Emerging trends include hybrid segmentation–classification architectures, transformer-based networks, and clinician-in-the-loop approaches. Determinants of performance encompass reference standard quality, dataset diversity, anatomical complexity, and adherence to STARD-AI and TRIPOD-AI reporting frameworks.

**Conclusions:**

AI demonstrates clinically relevant diagnostic accuracy in periodontal imaging, especially for measurement standardization and decision support. Although autonomous diagnosis remains premature, integrating explainable, externally validated AI systems within clinician-guided workflows supported by standardized reporting offers a practical route toward clinical translation.

## 1 Introduction

Periodontal diseases are among the most prevalent chronic inflammatory conditions globally, affecting billions of individuals and contributing substantially to the global burden of oral disease (1, 2). Accurate and early diagnosis is essential to prevent progression and tooth loss. Conventional diagnostic approaches rely primarily on clinical probing and two-dimensional (2D) radiographic interpretation, both of which have recognized limitations. Clinical measurements are prone to examiner variability and may underestimate localized defects, while 2D radiographs provide only planar projections that can distort anatomical relationships and obscure bone morphology (3–5).

Artificial intelligence (AI), particularly deep learning (DL) methods such as convolutional neural networks (CNNs), has emerged as a powerful adjunct to conventional diagnostic workflows in dentistry (6–9). Over the last decade, AI applications have rapidly expanded across dental disciplines, including periodontology, endodontics, caries detection, and implant planning. Various imaging modalities have been explored, including periapical and bitewing radiographs, panoramic radiographs, cone-beam computed tomography (CBCT), and intraoral photographs (8, 10–16). Across these modalities, AI has been applied to critical diagnostic tasks, such as detecting periodontal bone loss (PBL), measuring alveolar bone levels, identifying furcation involvement, and detecting periapical lesions.

On periapical radiographs, large-scale diagnostic studies have shown that CNN-based classifiers can achieve accuracies between 0.82 and 0.85, with AUC values above 0.88, often comparable to or exceeding average clinician performance (6, 12, 17). Systematic reviews and meta-analyses have affirmed the trend toward high diagnostic performance, and in individual AI imaging studies, specificities as high as 0.98 have been reported—underscoring the promise of AI-assisted periodontal diagnosis (15, 18, 19). In panoramic radiographs, CNN-based models have been reported to achieve diagnostic accuracies exceeding 90%, in some cases outperforming dentists whose accuracy ranges around 76%–78%, indicating balanced sensitivity–specificity profiles (20). However, despite these strong internal results, our DeepLabv3+ model demonstrated a notable decline in performance when evaluated on external data, reflecting a pattern widely observed in dental AI systems. For example, Chau et al. (21) reported high internal accuracy using a CNN-based photographic system for gingivitis detection, yet external validation of the same mHealth tool in Chau et al. (22) showed a substantial drop in specificity. Such discrepancies highlight the impact of dataset shift, arising from variations in imaging devices, acquisition protocols, and software versions; device heterogeneity across clinical sites; and differences in lighting conditions, angulation, and overall image quality, particularly in real-world dental imaging. Collectively, these factors contribute to external generalizability limitations, emphasizing the need for rigorous multi-center external validation before AI systems can be reliably deployed across diverse populations and imaging environments. Recent advancements have expanded to CBCT imaging, enabling volumetric assessments and detection of complex anatomical changes. Deep learning–based segmentation models have demonstrated promising performance, achieving accuracies around 0.91 and AUC values up to 0.96 for alveolar bone loss detection, with moderate performance in furcation defect identification (AUC ≈ 0.81). These findings highlight the potential of AI to enhance diagnostic precision in 3D periodontal imaging (13, 23). Intraoral photographic applications, increasingly relevant for screening and teledentistry, have shown variable diagnostic performance (with some studies reporting classification accuracies as low as ∼0.46, others approaching 1.00), though these extremes are not consistent across all works, owing to differences in protocol, device type, and reference standard (14, 24, 25).

Methodological developments have also matured. Frameworks such as STARD-AI and TRIPOD-AI provide standardized guidance for transparent reporting of AI-based diagnostic accuracy studies (25–28). Studies are increasingly incorporating external validation, multicenter datasets, and explainable AI techniques, aligning dental AI research with broader medical imaging standards (29). Recent dental CBCT/CT studies—for example in tooth segmentation meta-analyses, mandibular canal or root resorption quantification, and gender/age estimation using attention-based CBCT models—demonstrate this trend and show excellent performance in external/test datasets (30–34). Several recent overviews have synthesized AI performance across dental fields and highlighted the importance of structured reporting, external validation, and clinician engagement for successful clinical translation. Notably, Jundaeng et al. (35) review trends in AI for periodontitis diagnosis, and Tuygunov et al. (36) provide a comprehensive overview of challenges and enablers in integrating AI into dental practice (35, 36).

Given this rapidly expanding and heterogeneous evidence base, a thematic narrative review is warranted to synthesize current literature on AI diagnostic accuracy in periodontics. This review groups studies thematically by imaging modality, diagnostic task, and AI architecture, while examining determinants of performance and implications for clinical integration. This study was conducted as a thematic narrative review rather than a systematic review, and therefore did not follow a formal PRISMA-based screening or quantitative meta-analytic protocol.

## 2 Main text—thematic narrative review

### 2.1 Periapical and bitewing radiographs

Periapical and bitewing radiographs have been the most widely used modalities for AI applications in periodontal diagnostics, given their routine use in clinical practice and relatively high spatial resolution. CNN-based models have achieved diagnostic accuracies between 0.82 and 0.85 with AUC values above 0.88, comparable to average clinician performance (12, 15). An overview of the key characteristics and diagnostic performance of the included studies is summarized in Table 1. Similar performance trends have also been observed in panoramic radiographs (19). Subsequent studies have leveraged larger multicenter datasets, advanced segmentation–classification pipelines, and external validation strategies to enhance generalizability (37, 38). For example, Yadalam et al. (38) conducted a multicenter external validation of deep CNNs on periapical radiographs for alveolar bone loss detection, reporting sensitivities above 0.90 and consistent performance across centers (38).

**Table 1:** Summary of the 35 synthesized studies on AI diagnostic accuracy in periodontics (2019–2025).

No.	Author(s), Year	Imaging modality	Diagnostic task/focus	AI model/approach	Key findings (accuracy/AUC)	Ref.
1	AlGhaihab et al. (2025)	Periapical/Bitewing	Alveolar bone loss detection	Deep CNN	Accuracy >0.85; strong correlation with manual measures	(10)
2	Çelik et al. (2023)	Panoramic	Periapical lesion detection	CNN	Reliable lesion detection vs. clinicians	(11)
3	Hoss et al. (2023)	Periapical	Periodontal bone loss detection	Multiple CNNs	AUC >0.88; comparable to dentists	(12)
4	Tariq et al. (2023)	Systematic Review	Radiographic PBL detection	Meta-analysis	Pooled specificity 0.91–0.98	(15)
5	Iacob et al. (2025)	Systematic Review	PBL in 2D radiographs	Meta-analysis	Consistent high accuracy across CNNs	(18)
6	Kabir et al. (2021)	Periapical	Periodontitis stage grading	HYNETS hybrid CNN	AUC ≈0.97	(39)
7	Chang et al. (2020)	Periapical	Bone loss and staging	Hybrid DL model	Accuracy 0.94; improved reproducibility	(62)
8	Muhammed Sunnetci et al. (2022)	Periapical	Bone loss classification	Hybrid DL + ML	Improved interpretability	(63)
9	Dujic et al. (2023)	Periapical	PBL detection	Vision Transformer	High accuracy, good generalization	(64)
10	Mei et al. (2025)	Periapical	Disease diagnosis	Clinical knowledge-guided hybrid	Enhanced accuracy vs. baseline CNN	(37)
11	Widyaningrum et al. (2025)	Panoramic	Periodontitis detection/staging	Two-stage CNN	Accuracy 0.91–0.94	(40)
12	Chen et al. (2024)	Periapical	Early bone loss diagnosis	CNN	Accuracy 0.90; supports early detection	(41)
13	Krois et al. (2021)	Cross-modal	Dental image analysis	CNN generalizability	Model transferability validated	(42)
14	Kurt-Bayrakdar et al. (2025)	CBCT	Bone loss pattern detection	Deep CNN	Accuracy 0.91; volumetric precision	(13)
15	Xue et al. (2024)	Panoramic	Bone loss & periodontitis stage	DL classifier	Accuracy >0.84; AUC 0.92	(16)
16	Chatzopoulos et al. (2025)	Panoramic	Furcation defect classification	Systematic review	Highlights AI potential in furcation analysis	(20)
17	Shetty et al. (2024)	CBCT	Furcation involvement	CNN	Accuracy 0.91; AUC 0.98	(45)
18	Palkovics et al. (2025)	CBCT	Periodontal bone segmentation	DL segmentation	Accurate 3D bone contour detection	(50)
19	Pan et al. (2025)	CBCT	Mandibular canal localization	CNN	Robust multicenter generalization	(51)
20	Rashid & Gaghor (2025)	CBCT	Bone quantity assessment	DL quantification	Accurate cross-sectional measurements	(57)
21	Widiasri et al. (2023)	CBCT	Bone & canal segmentation	U-Net	High Dice score; reliable segmentation	(58)
22	Naufal et al. (2024)	CBCT	3D reconstruction	YOLOv8 segmentation	Sub-mm accuracy vs. reference	(56)
23	Zhang et al. (2025)	Panoramic	Furcation classification	Vision Transformer	AUC >0.95	(59)
24	Zhou et al. (2025)	CBCT	Tooth instance segmentation	Open DL framework	Improved cross-task transferability	(47)
25	Chen et al. (2023)	CBCT	Dental segmentation	CTA-UNet (CNN–Transformer)	Outperformed U-Net baseline	(48)
26	Zhao et al. (2025)	CBCT	Implant classification/segmentation	Multi-task learning	AUC 0.94; effective joint learning	(49)
27	Liu et al. (2023)	CBCT + Intraoral	Multimodal 3D fusion	DL fusion	Superior structural reconstruction	(46)
28	Mao et al. (2025)	Intraoral Photo	Periodontitis detection	Systematic review	AUC 0.80–0.93; variable standards	(14)
29	Tao et al. (2025)	Intraoral Photo	Periodontitis screening	DL photo processing	AUC 0.93; high sensitivity	(53)
30	Wen et al. (2024)	Intraoral Photo	Gingival inflammation grading	CNN with removal strategy	Accuracy 0.84–0.88	(54)
31	Felsch et al. (2023)	Intraoral Photo	Caries/hypomineralization detection	Vision Transformer	AUROC 0.93	(52)
32	Yadalam et al. (2025)	Periapical	External validation of bone loss classifier	Dual-embedding few-shot CNN	Sensitivity >0.90	(38)
33	Kot et al. (2025)	CBCT/CT	Tooth segmentation meta-analysis	Systematic meta-analysis	Pooled Dice ≈0.92	(32)
34	Baena-de la Iglesia et al. (2025)	CBCT	External root resorption quantification	AI-aided 3D analysis	High volumetric agreement	(30)
35	da Andrade-Bortoletto et al. (2025)	CBCT	Mandibular canal segmentation	Comparative validation	Accuracy >0.90	(31)

Hybrid deep learning models that first segment alveolar bone or tooth structures and then classify bone loss severity or disease stage have increasingly outperformed classification-only approaches, especially in quantification tasks. For example, HYNETS (Kabir et al.) reports AUC ≈ 0.97 for stage grading from periapical radiographs, using entangled segmentation and classification modules (39). More recently, Mei et al. (37) introduced a clinical knowledge–guided hybrid classification network combining localization and classification branches to improve periodontal diagnosis (37). Another work by Widyaningrum et al. (40) demonstrated a two-stage pipeline (detect → classify) for periodontal bone loss detection and staging (40). Explainability tools such as Grad-CAM and clinician-in-the-loop interfaces have enhanced interpretability and acceptance among practitioners' trust in AI-assisted periodontal diagnostics (7, 8, 23, 36). A recent systematic review found that AI-based PBL detection on periapical radiographs consistently achieved high specificity (0.91–0.98) and improved measurement reproducibility compared to manual assessments (18, 41, 42).

### 2.2 Panoramic radiographs and CBCT

Panoramic radiographs offer wide coverage but have inherent distortions and overlapping structures, challenging AI detection accuracy. CNN models trained on panoramic images have achieved accuracies around 0.80–0.84, with balanced sensitivity and specificity (13, 16, 20). Large dataset studies (e.g., Kurt-Bayrakdar et al. (13) show that deep learning models can approach or match clinician diagnostic performance for bone loss classification using panoramic radiographs, although performance drops in certain defect types such as vertical bone loss, areas in posterior regions, or where anatomical overlap is substantial (13, 16, 43).

CBCT provides volumetric information that allows AI models to evaluate complex periodontal structures in three dimensions. Recent CBCT-based investigations, including high-resolution CBCT texture-analysis work (71) have demonstrated that deep CNNs and advanced segmentation architectures can achieve high diagnostic performance across selected periodontal tasks. For instance, a ResNet101V2 model achieved approximately 0.91 test accuracy and an AUC of 0.98 for detecting furcation involvement on axial CBCT slices, while segmentation models have reported AUC values approaching 0.96 for total alveolar bone loss detection. Although performance varies across tasks and datasets, these findings highlight the strong potential of AI for volumetric periodontal assessment and underscore the need for larger, multicenter validation studies (13, 44, 45). Recent work has explored multimodal fusion, combining CBCT with other imaging sources. For example, a deep-learning fusion model integrating CBCT and intraoral mesh scans achieved superior accuracy for structural reconstruction, and frameworks combining CBCT+ panoramic registration have shown improved detection of furcation and alveolar bone patterns compared with single modalities (46, 47).

Recent CNN–transformer hybrid and multi-task frameworks have improved CBCT performance by integrating segmentation, classification and measurement into unified pipelines; examples include CTA-UNet and Swin/UNetR-based systems that leverage global attention for improved tooth/ bone segmentation and downstream classification (47–49). External-validation studies on multicenter CBCT datasets have demonstrated robust generalizability for anatomical localization and segmentation tasks (e.g., mandibular canal localization, periodontal defect segmentation), supporting cross-site deployment after appropriate validation (45, 50, 51).

### 2.3 Intraoral photographs

Intraoral photographic applications are gaining traction, especially for screening and teledentistry settings where radiographic facilities may be limited. Reported diagnostic accuracies vary widely (≈0.46–1.00), primarily because of inconsistent imaging protocols, variable lighting conditions, and heterogeneous reference standards (14). Recent studies using standardized imaging workflows and modern CNN/transformer or hybrid CNN-transformer architectures have improved performance in controlled settings—for example, a ResNet50-based multi-instance model reached an AUROC of 0.93 on internal and external tests for stage II–IV periodontitis, and DenseNet/CNN models have reported AUROCs around 0.80–0.84 for gingival inflammation grading—indicating that protocol standardization plus modern architectures can substantially increase photographic diagnostic accuracy (52–54).

A systematic review by Mao et al. (14) highlighted the promise of AI for photographic detection of periodontal disease but emphasized the urgent need for standardized acquisition protocols and multicenter datasets (14). Clinician-in-the-loop systems are particularly relevant here, allowing AI to serve as an assistive tool for remote triaging rather than as an autonomous diagnostic system.

### 2.4 Themes by diagnostic task

Grouping studies by diagnostic task provides insight into how AI performance varies according to the clinical endpoint:

Periodontal Bone Loss Detection: Most studies focus here, particularly on periapical and panoramic radiographs. CNNs and hybrid segmentation-classification architectures achieve high specificity (>0.90) and good sensitivity, especially for moderate-to-severe defects (12, 19, 40, 55).
Alveolar Bone Measurement: Multi-task deep learning models using YOLOv8 and ResNet backbones have demonstrated sub-millimeter error in alveolar crest measurement compared to CBCT gold standards (37, 56–58).
Furcation Involvement: CBCT-based CNNs and multimodal fusion approaches outperform panoramic models, achieving AUC values above 0.95 (13, 45, 59).
Periapical Lesion Detection: Transformer-based networks validated on multinational datasets showed superior sensitivity and external generalizability compared to baseline CNNs (17, 60, 61).

This task-based perspective underscores that AI performance is highest for volumetric and measurement tasks using CBCT, while 2D modalities remain highly useful for screening and routine clinical support.

### 2.5 Themes by AI architecture

Early studies relied mainly on standard CNN architectures (e.g., U-Net, ResNet) for either segmentation or classification. Over time, three major trends have emerged:

Segmentation–Classification Hybrids: Hybrid models combining segmentation and classification have improved bone contour localization and bone loss quantification, particularly in periapical radiographs. Notable examples include Chang et al. (62), Sunnetci et al. (63), Dujic et al. (64), and Widyaningrum et al. (40), which applied CNN-based hybrids for periodontal bone loss assessment, and the U-Net architecture (29) as a foundational segmentation framework.
Transformer-Based Models: Enhanced contextual understanding, particularly in panoramic and CBCT tasks, improving AUC by 3%–5% over CNN baselines (65–67).
Clinician-in-the-Loop & Explainable AI: Integration of Grad-CAM, attention maps, and uncertainty quantification to increase interpretability and user trust (68–70).

Recent methodological frameworks such as TRIPOD-AI and STARD-AI have influenced model development and reporting, encouraging external validation, clear reference standards, and transparent evaluation metrics (28, 71).

## 3 Discussion

Determinants of Diagnostic Performance:

Imaging Modality and Anatomical Complexity: CBCT provides volumetric information that enables superior AI performance in furcation detection and alveolar bone measurements, with accuracies up to 0.91 and AUC values above 0.95 (37). U-Net architectures (29) provide the foundational segmentation framework, while reporting standards such as TRIPOD-AI and STARD-AI (25, 26, 72) ensure transparent evaluation and external validation. Panoramic and intraoral photographs, by contrast, are more prone to distortions and variability, resulting in wider performance ranges (14, 36).

Dataset Diversity and External Validation: Earlier single-center studies often reported high internal accuracies that did not generalize when evaluated on external datasets. Recent validation work has reinforced this limitation. For example, Hadzic et al. (60) demonstrated that a periapical lesion detection CNN trained on one dataset showed reduced performance when tested on an independent, clinically representative CBCT dataset, underscoring the impact of real-world variability (60). More recent multicenter and cross-architecture investigations have emphasized similar issues. The comparative study by Schneider et al. (73), evaluating CNNs, transformers, and hybrid models across multiple institutions, showed that diagnostic accuracy and model calibration varied substantially when models were applied across heterogeneous datasets (73). Likewise, Yadalam et al. (38) highlighted the sensitivity of AI systems to dataset diversity, noting that their few-shot learning framework for bone-loss classification on OPG data remained highly dependent on training-domain characteristics and would require rigorous external validation for broader deployment (38). Collectively, these findings demonstrate that population differences, device heterogeneity, and broader domain-shift effects can significantly degrade performance, reinforcing the need for robust, multi-institutional external validation to ensure real-world applicability.

Reference Standards: Studies using CBCT or clinically validated measurements as reference standards consistently outperform those relying on single-expert or 2D radiographic assessments. The choice of gold standard directly impacts reported sensitivity and specificity, especially for alveolar bone measurements and furcation classification (18, 37, 41, 42).

Methodological Rigor and Reporting Quality: Adherence to frameworks such as STARD-AI and TRIPOD-AI has increased over the past two years, leading to more transparent study designs and reproducible results (26, 72). Explainability features and uncertainty quantification also play a role in clinician acceptance (23, 36).

### 3.1 Clinical implications

AI has significant potential to enhance periodontal diagnosis through standardized measurements, workflow efficiency, and decision support. High specificity in periapical radiograph applications may reduce inter-examiner variability and improve longitudinal monitoring in both specialist and general practice settings (12, 18, 19, 38, 41, 42). CBCT-based AI systems, with their volumetric capabilities, are especially suited for complex diagnostic tasks such as furcation involvement and surgical planning, providing quantitative metrics that complement clinician judgment (37). Foundational segmentation frameworks such as U-Net (29) support model development, while reporting standards like TRIPOD-AI and STARD-AI (25, 26, 72) ensure transparency and reproducibility.

For panoramic and intraoral photographic modalities, AI can serve as a screening or triaging tool, particularly in primary care and teledentistry contexts where access to CBCT is limited (14, 33). Clinician-in-the-loop models further ensure that AI outputs are interpreted within a professional diagnostic framework, reducing the risk of over-reliance on automated systems.

### 3.2 Policy and integration considerations

For AI to transition from research to clinical use, several policy and integration factors must be addressed:

Regulatory Alignment: AI diagnostic systems should align with regulatory frameworks (e.g., FDA, EMA, local dental boards) that emphasize transparency, explainability, and post-market surveillance.
Infrastructure and Data Governance: Successful clinical integration requires secure data pipelines, federated learning frameworks, and standardized imaging protocols to enable robust external validation.
Education and Workforce Integration: Dental education must include AI literacy to ensure clinicians can critically interpret AI outputs and integrate them into diagnostic reasoning.

These considerations are consistent with trends in medical imaging AI, where regulatory and infrastructural readiness significantly influences clinical adoption (26, 28, 68, 72).

## 4 Limitations

Despite rapid advancements, the evidence base remains limited by several factors:

Heterogeneity of Study Design: Differences in dataset size, anatomical sites, labeling strategies, and evaluation metrics complicate direct comparisons across studies.
Limited Prospective Trials: Most included studies are retrospective; few prospective or real-time clinical validation studies exist.
Variable Reporting Standards: Although STARD-AI and TRIPOD-AI adoption is improving, many studies still lack clear descriptions of preprocessing, validation, or error analysis.
Underrepresentation of Certain Populations: Multicenter datasets remain geographically concentrated, raising questions about generalizability to diverse patient groups.

Addressing these limitations will require coordinated methodological efforts, standardization, and regulatory oversight.

Beyond methodological constraints, several additional sources of bias must be considered in AI-assisted periodontal diagnosis. Dataset bias—including imbalances in disease prevalence, demographic distribution, and imaging conditions—can lead models to learn spurious associations rather than true pathological features. Reference-standard variability, particularly when ground-truth labels are derived from heterogeneous clinical judgments or non-standardized criteria, further undermines model reliability. Even clinician-provided annotations may lack consistency without calibration exercises, resulting in systematic differences in “ground-truth” labels that propagate into model errors. In conjunction with these factors, the risk of automation bias remains a major concern: clinicians may over-rely on algorithmic outputs, accept incorrect AI predictions, or overlook contradictory clinical evidence. This phenomenon has been documented in medical AI settings, where over-trust in automated systems can result in diagnostic complacency, reduced critical appraisal, and delayed error detection—especially when models encounter out-of-distribution cases (69–71). Implementing clinician-in-the-loop workflows, standardized labeling protocols, calibration sessions for annotators, and explainable AI interfaces is therefore essential to mitigate these risks and preserve clinical accountability in AI-supported periodontal decision-making.

### 4.1 Future directions

Future research should prioritize:

Multicenter Prospective Clinical Trials using standardized protocols and external validation to evaluate AI under real-world conditions.
Task-Specific Architectures, including transformer-based multi-task networks, to improve performance on complex volumetric tasks.
Explainable and Uncertainty-Aware AI, fostering clinician trust and regulatory compliance.
Integration of AI into Longitudinal Care, enabling dynamic tracking of periodontal changes over time and personalized treatment planning.

These directions align with broader trends in AI for medical imaging and offer a roadmap for meaningful clinical integration in periodontics.

## 5 Conclusions

Artificial intelligence has progressed rapidly in periodontal diagnostics over the past five years, moving from proof-of-concept studies to externally validated, multicenter applications across multiple imaging modalities. CNN-based systems for periapical radiographs consistently achieve high specificity and accuracy, supporting their use for standardized measurements and clinical decision support. CBCT-based models offer the highest diagnostic performance for volumetric tasks, including alveolar bone measurement and furcation involvement detection, while panoramic and intraoral photographic applications provide valuable tools for screening and triage, particularly in primary care and teledentistry settings.

Despite these advances, methodological heterogeneity, limited prospective evidence, and variable reporting standards remain barriers to widespread clinical adoption. The integration of reporting frameworks such as STARD-AI and TRIPOD-AI, combined with explainable AI techniques and clinician-in-the-loop workflows, offers a realistic pathway toward safe and generalizable deployment.

Future efforts should focus on multicenter prospective trials, regulatory alignment, and standardized imaging protocols to ensure robust, equitable, and clinically meaningful integration of AI systems into periodontal practice. AI should be viewed not as a replacement for clinician expertise but as a complementary tool that enhances diagnostic accuracy, consistency, and patient care.
