# Supplementary material for: Accuracy of artificial intelligence applications in periodontics: a thematic narrative review
Source: Front Dent Med. 2026 Jan 22;7:1729825. doi: 10.3389/fdmed.2026.1729825 (PMC12872754; doi:10.3389/fdmed.2026.1729825)
Supplement: Supplementary file 1 [file Datasheet1.pdf]

Table 1. Summary of the 35 Synthesized Studies on AI Diagnostic Accuracy in Periodontics (2019–2025).

**No.**	**Author(s), Year**	**Imaging Modality**	**Diagnostic Task / Focus**	**AI Model / Approach**	**Key Findings (Accuracy / AUC)**	**Ref.**
1	AlGhaihab A et al., 2025	Periapical/Bitewing	Alveolar bone loss detection	Deep CNN	Accuracy >0.85; strong correlation with manual measures	[10]
2	Çelik B et al., 2023	Panoramic	Periapical lesion detection	CNN	Reliable lesion detection vs clinicians	[11]
3	Hoss P et al., 2023	Periapical	Periodontal bone loss detection	Multiple CNNs	AUC >0.88; comparable to dentists	[12]
4	Tariq A et al., 2023	Systematic Review	Radiographic PBL detection	Meta-analysis	Pooled specificity 0.91–0.98	[15]
5	Iacob AM et al., 2025	Systematic Review	PBL in 2D radiographs	Meta-analysis	Consistent high accuracy across CNNs	[18]
6	Kabir T et al., 2021	Periapical	Periodontitis stage grading	HYNETS hybrid CNN	AUC ≈0.97	[36]
7	Chang HJ et al., 2020	Periapical	Bone loss and staging	Hybrid DL model	Accuracy 0.94; improved reproducibility	[60]
8	Sunnetci K et al., 2022	Periapical	Bone loss classification	Hybrid DL + ML	Improved interpretability	[61]

9	Dujic H et al., 2023	Periapical	PBL detection	Vision Transformer	High accuracy, good generalization	[62]
10	Mei L et al., 2025	Periapical	Disease diagnosis	Clinical knowledge-guided hybrid	Enhanced accuracy vs baseline CNN	[34]
11	Widyaningrum R et al., 2025	Panoramic	Periodontitis detection/staging	Two-stage CNN	Accuracy 0.91–0.94	[37]
12	Chen IH et al., 2024	Periapical	Early bone loss diagnosis	CNN	Accuracy 0.90; supports early detection	[38]
13	Krois J et al., 2021	Cross-modal	Dental image analysis	CNN generalizability	Model transferability validated	[39]
14	Kurt-Bayrakdar S et al., 2025	CBCT	Bone loss pattern detection	Deep CNN	Accuracy 0.91; volumetric precision	[13]
15	Xue T et al., 2024	Panoramic	Bone loss & periodontitis stage	DL classifier	Accuracy >0.84; AUC 0.92	[16]
16	Chatzopoulos GS et al., 2025	Panoramic	Furcation defect classification	Systematic review	Highlights AI potential in furcation analysis	[19]
17	Shetty S et al., 2024	CBCT	Furcation involvement	CNN	Accuracy 0.91; AUC 0.98	[42]
18	Palkovics D et al., 2025	CBCT	Periodontal bone segmentation	DL segmentation	Accurate 3D bone contour detection	[47]

19	Pan X et al., 2025	CBCT	Mandibular canal localization	CNN	Robust multicenter generalization	[48]
20	Rashid MO & Gaghor S, 2025	CBCT	Bone quantity assessment	DL quantification	Accurate cross-sectional measurements	[54]
21	Widiasri M et al., 2023	CBCT	Bone & canal segmentation	U-Net	High Dice score; reliable segmentation	[55]
22	Naufal M et al., 2024	CBCT	3D reconstruction	YOLOv8 segmentation	Sub-mm accuracy vs reference	[53]
23	Zhang X et al., 2025	Panoramic	Furcation classification	Vision Transformer	AUC >0.95	[56]
24	Zhou Y et al., 2025	CBCT	Tooth instance segmentation	Open DL framework	Improved cross-task transferability	[44]
25	Chen Z et al., 2023	CBCT	Dental segmentation	CTA-UNet (CNN–Transformer)	Outperformed U-Net baseline	[45]
26	Zhao Y et al., 2025	CBCT	Implant classification/segmentation	Multi-task learning	AUC 0.94; effective joint learning	[46]
27	Liu J et al., 2023	CBCT + Intraoral	Multimodal 3D fusion	DL fusion	Superior structural reconstruction	[43]
28	Mao K et al., 2025	Intraoral Photo	Periodontitis detection	Systematic review	AUC 0.80–0.93; variable standards	[14]

29	Tao LR et al., 2025	Intraoral Photo	Periodontitis screening	DL photo processing	AUC 0.93; high sensitivity	[50]
30	Wen C et al., 2024	Intraoral Photo	Gingival inflammation grading	CNN with removal strategy	Accuracy 0.84–0.88	[51]
31	Felsch M et al., 2023	Intraoral Photo	Caries/hypomineralization detection	Vision Transformer	AUROC 0.93	[49]
32	Yadalam PK et al., 2025	Periapical	External validation of bone loss classifier	Dual-embedding few-shot CNN	Sensitivity >0.90	[35]
33	Kot WY et al., 2025	CBCT/CT	Tooth segmentation meta-analysis	Systematic meta-analysis	Pooled Dice ≈0.92	[29]
34	Baena-de la Iglesia T et al., 2025	CBCT	External root resorption quantification	AI-aided 3D analysis	High volumetric agreement	[27]
35	da Andrade-Bortoletto MFS et al., 2025	CBCT	Mandibular canal segmentation	Comparative validation	Accuracy >0.90	[28]
